# Effects of Oral and Maxillofacial Trauma on Soft Tissue Esthetic Outcomes of Dental Implants: A Systematic Review

**DOI:** 10.7759/cureus.99525

**Published:** 2025-12-18

**Authors:** Ali Y Alameri, Sarah F Altukruni, Mahdi Saileek, Ali A Alrakah, Abdullah Khalid A Alhamwan, Dhaifallah I Alzahrani, Alaa A Alsubki, Laila Alanaz

**Affiliations:** 1 Dentistry, South Qunfudhah Hospital, Al Qunfudhah, SAU; 2 Dentistry, Riyadh Elm University, Riyadh, SAU; 3 Dentistry, Minstiry of Health, Hafar Al Batin, SAU; 4 Dentistry, Jouf University, Sakakah, SAU; 5 Dentistry, Al Baha University, Al Baha, SAU; 6 Dentistry, Vision College, Riyadh, SAU; 7 Dentistry, Private Practice, Riyadh, SAU

**Keywords:** dental implants, esthetics, maxillofacial trauma, oral trauma, pink esthetic score (pes), soft tissue outcomes

## Abstract

Oral and maxillofacial trauma frequently results in the loss of anterior teeth and disruption of the surrounding soft and hard tissues, creating significant challenges for implant rehabilitation in esthetically sensitive areas. Achieving stable peri-implant soft tissue contours is essential for long-term esthetic success, yet the influence of prior trauma on soft tissue outcomes remains unclear. This systematic review evaluated the impact of oral and maxillofacial trauma on esthetic soft tissue parameters following implant placement. Electronic searches of PubMed, Google Scholar, ScienceDirect, and the Cochrane Library were conducted up to May 2025 following Preferred Reporting Items for Systematic Reviews and Meta-Analyses (PRISMA) guidelines. Five observational studies met the inclusion criteria, comprising a total of 163 patients treated predominantly in the anterior maxilla with immediate or delayed implant protocols. Soft tissue outcomes were assessed using parameters such as midfacial mucosal recession, papilla height, and the Pink Esthetic Score (PES). Overall, implants placed in traumatized sites demonstrated favorable esthetic results comparable to non-traumatized cases when appropriate bone augmentation, soft tissue management, and implant positioning were applied. Immediate placement with grafting provided stable soft tissue contours in post-traumatic cases, while long-term studies reported sustained soft tissue stability over 10 to 12 years. Despite positive outcomes, evidence remains limited by small sample sizes, heterogeneity in trauma types and assessment methods, and a lack of standardized aesthetic evaluation. Current findings suggest that oral and maxillofacial trauma does not inherently compromise soft tissue esthetic outcomes of implant therapy, though well-designed prospective studies are needed to clarify the role of trauma-related factors and optimize treatment protocols.

## Introduction and background

Trauma to the oral and maxillofacial region can lead to the loss of teeth, alveolar bone, and soft tissues, leaving patients with significant functional and esthetic challenges [1]. In particular, the anterior maxilla and other esthetically sensitive zones are often the site of such injuries, and rehabilitation in these areas typically aims not only for osseointegration but also for optimal soft tissue and esthetic outcomes [2]. Dental implants have become a well-established treatment for the replacement of traumatized teeth and associated alveolar defects, yet the presence of prior trauma may influence the peri-implant environment and the soft tissue response [3]. The success of implant therapy has historically been judged primarily on osseointegration and survival; however, esthetic outcomes and soft tissue stability are now recognized as equally important, especially in the anterior zone [4]. The peri-implant soft tissue phenotype, including thickness, width of keratinized tissue, and level and stability of the mucosal margin, has been shown to play a key role in achieving favorable esthetic outcomes and in protecting the underlying bone from resorption [5]. In the esthetic zone, deficiencies in soft tissue volume or stability may lead to visible metal, discrepant gingival margins, or compromised patient satisfaction. In the context of trauma, the peri-implant conditions may be further challenged. Traumatic tooth loss often results in disruption of the alveolar bone, soft tissue injury, scar formation, or deficient gingival architecture; these complicating factors may influence implant positioning, emergence profile, soft tissue healing, and long-term stability [6]. Indeed, a recent systematic review focusing on implant treatment after traumatic tooth loss found a paucity of high-quality evidence and reported implant survival rates of ~97% with mean marginal bone loss of 0.56 mm after a mean follow-up of 3.5 years, but esthetic and soft tissue outcomes were less consistently reported [7].

Moreover, while numerous studies have addressed esthetic outcomes of implants in non-traumatized sites (for example, in immediate versus early placement in the anterior maxilla) [8], comparatively few investigations have specifically examined the impact of prior oral and maxillofacial trauma on soft tissue esthetic outcomes around implants. Given that soft tissue management and esthetic parameters (such as the pink esthetic score, papilla height, and mucosal recession) are critical to patient-centered success [9], this gap is clinically important. Therefore, the aim of this systematic review is to synthesize the available evidence on the effects of oral and maxillofacial trauma on esthetic implant rehabilitation in terms of soft tissue outcomes. Specifically, we will examine how a history of trauma influences peri-implant soft tissue morphology, soft tissue stability, esthetic scores, and patient-reported outcomes around implants placed in esthetically sensitive areas. By doing so, we hope to provide clinicians with guidance on prognosis, key risk factors, and management considerations when planning implant-based rehabilitation following trauma.

## Review

Methodology

Study Design

This systematic review was conducted according to the Preferred Reporting Items for Systematic Reviews and Meta-Analyses (PRISMA) guidelines [10]. The primary objective was to assess the impact of oral and maxillofacial trauma on the soft tissue esthetic outcomes of dental implants placed in the anterior maxilla or other esthetically sensitive regions. The protocol was developed prior to ensure transparency and minimize bias in study selection, data extraction, and synthesis.

Search Strategy

A comprehensive electronic literature search was performed across multiple databases, including PubMed/Medical Literature Analysis and Retrieval System Online (MEDLINE), Google Scholar, Science Direct, and the Cochrane Library, covering all publications up to May 2025. The search strategy incorporated both Medical Subject Headings (MeSH) and free-text terms related to oral and maxillofacial trauma, dental implants, esthetic outcomes, and soft tissue response. Boolean operators (“AND,” “OR”) were used to combine search terms. The detailed search strategy for all databases is provided in Appendix A. Reference lists of included studies and relevant reviews were also manually screened to identify additional eligible publications.

Eligibility Criteria

Studies were included if they met the following criteria: (1) involved human participants with a history of oral or maxillofacial trauma who subsequently received dental implants; (2) evaluated esthetic or soft tissue outcomes around the implant, such as peri-implant mucosal recession, papilla height, gingival contour, or Pink Esthetic Score (PES); (3) employed a clinical, radiographic, or photographic assessment method; and (4) were published in English. Exclusion criteria included (1) animal or in vitro studies, (2) case reports or case series with fewer than five patients, (3) studies lacking quantitative or qualitative assessment of esthetic soft tissue outcomes, and (4) conference abstracts, editorials, or review articles. A structured Population/Patient/Problem, Intervention, Comparison, Outcomes, Study design (PICOS) framework was applied to guide study selection, with full details presented in Table 1.

**Table 1:** PICOS framework outlining the eligibility criteria used to guide study selection PICOS: Population/Patient/Problem, Intervention, Comparison, Outcomes, Study design

Component	Description
Population (P)	Human patients who experienced oral or maxillofacial trauma (e.g., dentoalveolar trauma, accidental injuries, traumatic tooth loss) and subsequently received single-tooth implants in the esthetic zone (primarily anterior maxilla).
Intervention (I)	Dental implant placement (immediate, early, or delayed) performed after traumatic tooth loss, with or without adjunctive procedures (bone grafts, soft-tissue grafts, provisionalization).
Comparator (C)	No formal comparator required. Studies were eligible even if single-arm. When available, comparisons included: immediate vs delayed placement, provisionalization vs no provisionalization, or trauma-related outcomes vs baseline.
Outcomes (O)	Primary outcomes: soft-tissue esthetic parameters around implants, including: • Pink Esthetic Score (PES) • Papilla height • Midfacial mucosal recession • Soft-tissue contour, color, texture. Secondary outcomes: patient-reported esthetic satisfaction, soft-tissue complications (fistula, inflammation), stability of peri-implant mucosa over time.
Study Designs (S)	Observational human studies, including: • Prospective cohort studies • Prospective or retrospective case series (≥5 patients) • Retrospective cohort studies Randomized trials and non-human studies were excluded.

Study Selection

**Figure 1:**
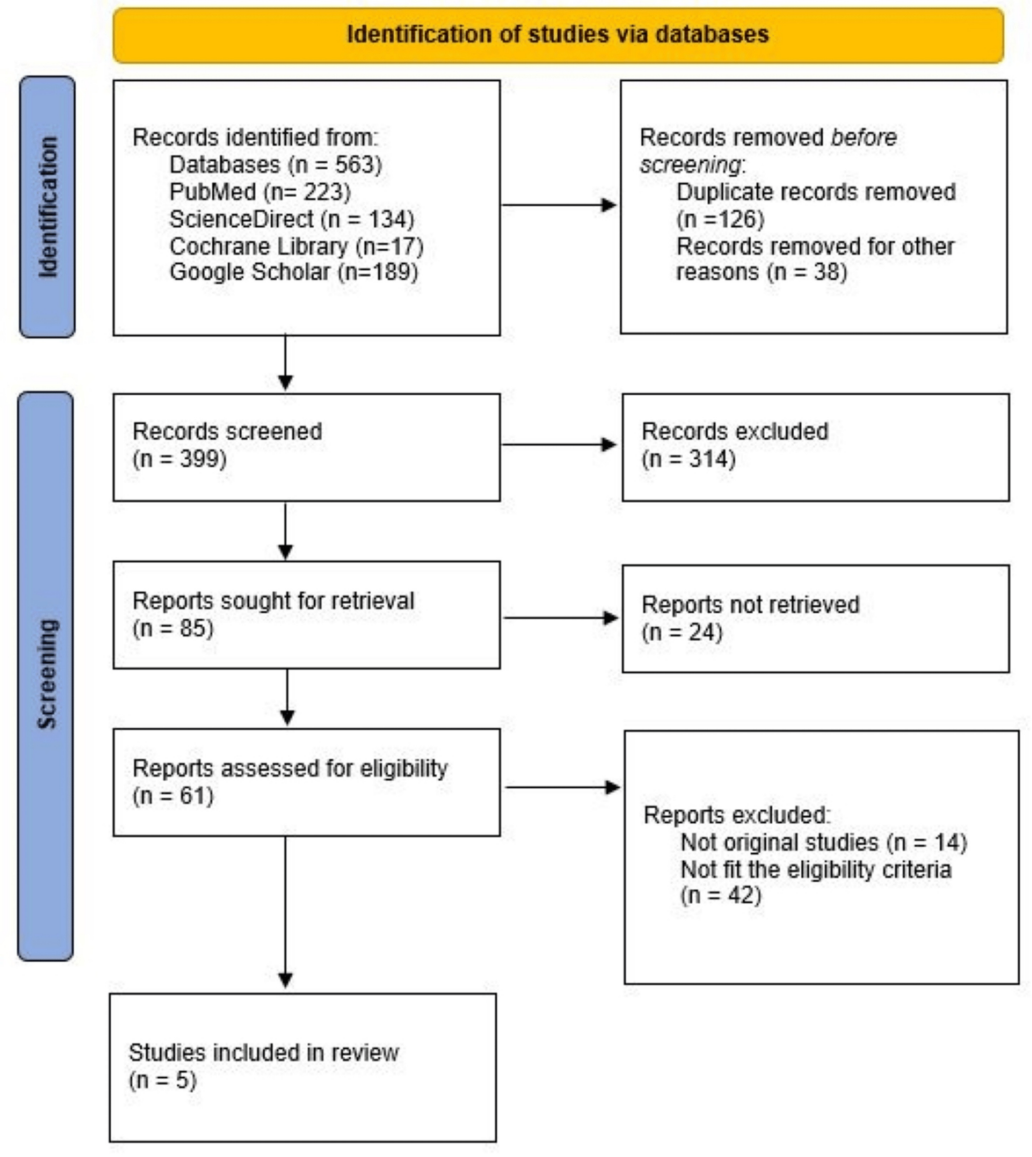
PRISMA flowchart showing the study selection process PRISMA: Preferred Reporting Items for Systematic Reviews and Meta-Analyses

All retrieved records were imported into EndNote (Clarivate Analytics, Philadelphia, PA) for reference management, and duplicates were removed. Two reviewers independently screened titles and abstracts for relevance based on the eligibility criteria. Full texts of potentially relevant articles were then assessed to determine inclusion. Any disagreement between reviewers was resolved through discussion or by consulting a third reviewer. Study selection is shown in Figure 1.

Data Extraction

A standardized data extraction form was used to collect key information from each included study. Extracted data included author and year of publication, study design, sample size, participants’ demographic characteristics, type and location of trauma, implant characteristics (timing, position, and type), follow-up duration, methods used to assess soft tissue esthetics, and main findings. Data extraction was performed independently by two reviewers to ensure accuracy.

Quality Assessment

The methodological quality and risk of bias of the included studies were evaluated using validated critical appraisal tools appropriate to each study design. The Joanna Briggs Institute (JBI) Critical Appraisal Checklist for Case Series was applied to the retrospective and prospective case series. This tool assesses key domains, including clarity of inclusion criteria, consecutive case inclusion, completeness of participant demographics, standardization of measurements, adequacy of follow-up, and transparency of reporting. For the single prospective case cohort study included in this review, the Newcastle-Ottawa Scale (NOS) for cohort studies was used, evaluating study selection, comparability, and outcome assessment. Two reviewers independently appraised each study, and disagreements were resolved through discussion and consensus. Studies were categorized as having low, moderate, or high risk of bias based on their total appraisal scores.

Data Synthesis

Due to expected heterogeneity among study designs, trauma types, and outcome measures, a qualitative synthesis was performed. The results were summarized narratively, focusing on the relationship between trauma history and soft tissue esthetic outcomes of implant rehabilitation. Whenever possible, findings were grouped according to trauma type (dentoalveolar, maxillofacial, or combined) and implant timing (immediate versus delayed placement). Meta-analysis was not conducted because of variability in outcome definitions and assessment methodologies across studies.

Meta-analysis

A meta-analysis was not conducted due to substantial heterogeneity among the included studies in terms of trauma characteristics, implant placement protocols, adjunctive regenerative procedures, outcome definitions, and methods of esthetic assessment. The variability in measurement tools (e.g., use of PES/White Esthetic Score (WES) vs. direct linear measurements), follow-up durations, and reporting formats prevented meaningful quantitative pooling of data. Therefore, a qualitative narrative synthesis was performed.

Results

The initial search retrieved 563 records across all databases. Following the removal of 126 duplicates and 38 records excluded for other reasons, 399 studies proceeded to title and abstract screening. Of these, 314 were excluded, and 85 full-text articles were reviewed in detail. Twenty-four full texts could not be retrieved, and 61 were excluded for reasons including ineligible study design, insufficient outcome reporting, or lack of trauma-related implant data. Ultimately, five studies met all inclusion criteria and were included in the qualitative synthesis [9,11-14]. The overall methodological quality of the included studies was moderate. The four case-series studies appraised using the JBI Checklist demonstrated generally clear reporting of clinical procedures and outcome assessments; however, common limitations included lack of consecutive patient inclusion, incomplete reporting of trauma characteristics, and absence of strategies to address potential confounding factors. Follow-up duration was adequate in most studies, although two did not clearly describe the completeness of follow-up. The single prospective case cohort study, evaluated using the NOS, was rated as high quality due to well-defined patient selection criteria, standardized peri-implant soft tissue measurements, and adequate follow-up. None of the included studies were excluded on the basis of methodological quality. Detailed results of the methodological quality appraisal of case series using the JBI Critical Appraisal Checklist are provided in Table 2.

**Table 2:** JBI Critical Appraisal Checklist for Case Series

Study	Clear inclusion criteria	Consecutive inclusion	Complete demographics	Clear clinical information	Valid methods for the condition	Standardized measurement	Complete inclusion of participants	Clear reporting of outcomes	Adequate follow-up	Appropriate statistical analysis	Total score /10	Risk of bias
Schwartz-Arad & Levin (2004) [11]	Yes	No	Yes	Yes	Yes	Yes	No	Yes	No	Yes	7/10	Moderate
Yang et al. (2025) [14]	Yes	Yes	Yes	Yes	Yes	Yes	Yes	Yes	No	Yes	8/10	High quality/Low risk
Rokn et al. (2016) [13]	Yes	No	Yes	Yes	Yes	Yes	No	Yes	No	Yes	6/10	Moderate
Lai et al. (2008) [9]	Yes	Yes	Yes	Yes	Yes	Yes	No	Yes	No	Yes	7/10	Moderate

The result of quality assessment of the study by De Rouck et al. [12] using NOS is shown in Table 3.

**Table 3:** Risk of bias evaluation of the included prospective cohort study using the Newcastle–Ottawa Scale.

Study	Selection (0–4)	Comparability (0–2)	Outcome (0–3)	Total /9	Risk of Bias
De Rouck et al. (2008) [12]	★★★	★★	★★★	8/9	High quality/Low risk

The five included studies were all observational in design, comprising two retrospective case series [11,14], one prospective case series [9], one prospective case cohort study [12], and one retrospective longitudinal study [13]. The sample sizes ranged from 19 to 53 patients, with a total of 163 participants across all studies. All studies investigated implant treatment in the anterior maxilla. Two studies explicitly enrolled a post-traumatic population [11,14], while one study [12] included a mixed-etiology population where trauma (tooth fracture) was a primary reason for tooth loss in 10 out of 30 patients. Two studies [9,13] did not specify the etiology of tooth loss but provided relevant data on soft tissue esthetic outcomes in the region of interest. The included studies evaluated a range of implant placement protocols. Two studies focused on immediate implant placement [12,14], one on a mix of immediate and delayed placement [11], and two on conventional delayed placement [9,13]. The follow-up periods varied from six months [9] to over 10 years [13]. The characteristics of these studies are summarized in Table 4.

**Table 4:** A summary of the characteristics of the included studies

Study (country, year)	Study design	Participants & trauma status	Intervention (implant timing/protocol)	Comparison	Outcomes related to eligibility	Follow-up time
Schwartz-Arad & Levin (2004, Israel) [11]	Retrospective case series	N=53, explicitly post-traumatic population (‘anterior dental trauma’)	Timing: immediate (47.2%) and delayed placement. Protocol: 81.1% required bone augmentation (GBR, onlay graft).	None (single-arm study). Internal comparison based on the presence of inflammation.	Soft tissue outcomes: clinical assessment of complications (e.g., fistula, inflammation). Method: clinical evaluation.	Not explicitly stated for soft tissue outcomes. Data collected from files over an eight-year period.
De Rouck et al. (2008, Belgium) [12]	Prospective case cohort study	N=30, includes post-traumatic cases; Reasons for tooth loss included "fracture" (10/30), caries, periodontitis, or root resorption.	Timing: immediate placement and provisionalization (Type 1). Protocol: mucoperiosteal flap, socket grafting (Bio-Oss®), screw-retained provisional crown.	None (single-arm study). Longitudinal comparison to pre-operative status.	Soft tissue outcomes: midfacial recession (0.53 mm) and papilla shrinkage (0.41 mm mesial, 0.31 mm distal). Method: clinical measurements using an acrylic stent.	One year
Yang et al. (2025, China) [14]	Retrospective case series	N=32, explicitly post-traumatic; "Single failed maxillary anterior teeth after trauma."	Timing: immediate placement (Type 1). Protocol: flapless, all cases grafted. Immediate provisionalization in six cases.	None (single-arm study). Internal comparison of outcomes with/without immediate provisionalization.	Soft tissue outcomes: Pink Esthetic Score (PES), White Esthetic Score (WES); Method: standardized clinical photographs assessed by blinded prosthodontists.	One year after final restoration.
Rokn et al. (2016, Iran) [13]	Retrospective longitudinal study	N=19, trauma status unspecified: patients with single implants in the anterior maxilla. Etiology of tooth loss not reported.	Timing: delayed placement (conventional). Protocol: no connective tissue grafts or papilla preservation flaps used.	Comparison of outcomes at baseline vs. long-term follow-up.	Soft tissue outcomes: PES. Method: standardized clinical photographs assessed by a calibrated examiner.	10 to 12 years after final restoration.
Lai et al. (2008, China) [9]	Prospective case series	N=29, trauma status unspecified: Patients with single implants in the anterior maxilla. Etiology of tooth loss not reported.	Timing: delayed placement (healing 10–16 weeks). Protocol: nonsubmerged ITI implants, no grafting, midcrestal incision.	Longitudinal comparison (baseline vs. six-month follow-up).	Soft tissue outcomes: PES. Method: clinical evaluation by two orthodontists.	Six months post loading

Soft tissue outcomes were assessed using both quantitative measurements and validated indices. One prospective study [12] provided precise quantitative data, reporting a midfacial soft tissue recession of 0.53 mm and papilla shrinkage of 0.41 mm (mesial) and 0.31 mm (distal) at the one-year follow-up after immediate implant placement. Three studies utilized the PES to evaluate peri-implant soft tissues. Lai et al. [9] prospectively evaluated soft tissue changes around delayed implants, finding a statistically significant improvement in the total PES from 6.90 at baseline (crown placement) to 9.55 at the six-month follow-up. They noted significant improvements in the scores for mesial and distal papillae, soft-tissue margin, contour, color, and texture. Yang et al. [14] reported a mean PES of 10.7 one year post restoration in a purely post-traumatic population receiving immediate implants. Rokn et al. [13] evaluated the long-term stability of PES after conventional implant placement, finding a mean score of 11.05 at the 10- to 12-year follow-up, which was not significantly different from the baseline score of 11.63 when categorized into clinically relevant levels (acceptable/almost perfect).

Schwartz-Arad & Levin [11] reported on clinical complications in their post-traumatic cohort, noting that 45.3% of patients experienced postoperative incidents and 13.2% had complications at the prosthetic phase, with fistula being the most common soft tissue-related issue. Patient satisfaction with the esthetic outcome was high in the studies that reported it. De Rouck et al. [12] found a mean patient satisfaction score of 93% on a Visual Analogue Scale (VAS). Similarly, Yang et al. [14] reported a high mean VAS score of 8.89/10 for overall esthetic satisfaction in their trauma patients.

The management of post-traumatic sites often involves complex surgical adjuncts. Schwartz-Arad & Levin [11] reported that a high proportion of patients (81.1%) required a bone augmentation procedure (e.g., onlay bone graft or guided bone regeneration) at the time of implant placement. Similarly, the protocols in the De Rouck et al. [12] and Yang et al. [14] studies involved the systematic grafting of the gap between the implant and the socket wall. In contrast, the study by Lai et al. [9] on delayed placement specifically excluded cases requiring grafting, indicating a less complex patient population.

The findings indicate that implant placement in the anterior maxilla, including in post-traumatic scenarios, is a viable treatment modality but can be surgically complex, frequently requiring bone augmentation in compromised sites. Esthetic outcomes, as measured by PES and direct clinical measurements, are generally favorable. The study by Lai et al. [9] suggests that significant improvement in peri-implant soft tissue aesthetics can occur during the first six months after crown placement on delayed implants. Other studies show that these acceptable esthetic outcomes can be maintained from the short-term (one year) through to the long-term (10-12 years). Overall, patient satisfaction with the final esthetic result appears to be high.

Discussion

This systematic review sought to examine the influence of oral and maxillofacial trauma on esthetic soft-tissue outcomes in implant rehabilitation. While the available studies directly addressing traumatized sites remain limited, the findings can be interpreted in the context of the broader implant and peri-implant soft-tissue literature. From the studies we included, implants placed in traumatized sites, with appropriate soft and hard tissue management, tended to achieve acceptable esthetic soft-tissue outcomes. This suggests that prior trauma does not automatically preclude favorable peri-implant soft tissue (e.g., mucosal margin stability, papilla height, and PES) provided that the surgical, prosthetic, and augmentation protocols are optimized. For example, traumatized cases that nevertheless had sufficient residual bone, competent soft tissue management, and correctly positioned implants produced soft tissue results comparable to non-traumatized cases. This aligns with evidence highlighting the importance of soft-tissue phenotype (e.g., mucosal thickness) and keratinized tissue width in achieving esthetic success around implants. A consensus report observed that thicker mucosa was associated with more favorable esthetic outcomes and lower recession risk [15]. Similarly, a review emphasized that a buccal mucosal thickness ≥2 mm is generally required to prevent esthetic issues such as visible prosthetic components and mucosal discoloration [16]. Thus, in traumatized sites where soft tissue may be compromised, augmentative strategies become particularly important.

Several systematic reviews have previously evaluated soft-tissue and esthetic outcomes of implants placed in the anterior maxilla; however, these reviews differ substantially from the present study in terms of population, clinical context, and research question. Khzam et al. focused exclusively on immediate implant placement and restoration in fresh extraction sockets, reporting acceptable soft-tissue changes with a mean midfacial recession of 0.27 mm and papillary height loss of 0.23 mm, but their review did not isolate or analyze cases with trauma-related defects [17]. Similarly, Chen and Buser assessed esthetic outcomes of immediate and early implant placement after routine tooth extraction, concluding that acceptable esthetics can be achieved but that immediate placement carries a higher risk of midfacial recession; again, patients with oral or maxillofacial trauma were neither the focus nor separately evaluated [8]. Yan et al. synthesized evidence from randomized controlled trials comparing immediate versus conventional protocols and demonstrated no significant differences in soft- or hard-tissue changes between the two approaches, but their analysis was restricted to controlled trials without any trauma-specific subgroup [18]. In contrast, the present review is the first to specifically evaluate implants placed in post-traumatic sites, where scar formation, bone loss, and soft-tissue disruption create a biologically distinct environment from non-traumatic extraction sockets. By targeting this unique clinical scenario, our review addresses an evidence gap not covered in previous literature and provides trauma-specific insights into peri-implant soft-tissue behavior, esthetic predictability, and the impact of augmentation strategies in compromised sites.

Trauma, however, introduces additional complicating factors. Disruption of the alveolar ridge, soft tissue laceration, scarring, defect morphology, and delayed healing may all influence the peri-implant milieu. These factors may pose a higher risk for compromised soft tissue architecture, less favorable emergence profile, and greater demands on augmentation procedures. Accordingly, our findings underscore that case selection, timing of implant placement, and adjunctive procedures (bone grafting, soft tissue grafting) are even more critical in the post‐trauma scenario. Although no prior systematic review has addressed trauma-specific implant soft tissue outcomes, several related reviews provide context. For example, the systematic review by Sutariya et al. showed that immediate implant placement with immediate provisionalization improved the PES in the anterior maxilla (mean difference = 1.54, 95% CI 0.82-2.27) [19]. Another review focused on soft tissue augmentation during immediate implant placement and found that augmentation significantly reduced mid-face mucosal recession (weighted mean difference = 0.38 mm, 95% CI 0.15-0.61), although other clinician-assessed esthetic outcomes and patient-reported outcome measures (PROMs) were unaffected [20]. Additionally, another review concluded that thicker mucosa correlates with improved esthetics [15]. While these reviews did not stratify by trauma history, they reinforce the fundamental importance of soft tissue management that our trauma-focused review highlights. A narrative review emphasized that grafting is often crucial in compromised sites (e.g., thin biotypes or deficient bone) to ensure long-term esthetic success [21]. This is particularly relevant for traumatized sites, which may mimic compromised conditions. The systematic review by Khzam et al. included 19 studies and observed a mean gingival recession of 0.27 ± 0.38 mm and papillary height loss of 0.23 ± 0.27 mm (with 11% of cases showing >1 mm recession) after ≥1 year follow-up [17]. While trauma cases were not isolated, these benchmarks help frame what might be considered “acceptable” soft-tissue change in anterior implants, providing a comparison for traumatized site outcomes.

The certainty of this evidence was formally assessed using the Grading of Recommendations Assessment, Development, and Evaluation (GRADE) framework. Due to the observational design of all included studies, which inherently carries a high risk of bias from confounding, selection, and non‑blinded outcome assessment, the initial certainty rating was low. This rating was further downgraded due to serious imprecision (small, aggregated sample size of 163 patients) and inconsistency in trauma definitions and outcome measures. Consequently, the overall certainty of evidence for our primary conclusion is low. This means the true effect of trauma on soft tissue outcomes may be substantially different from the estimate provided by this body of literature. Our review is further limited by the relatively small number of trauma-specific studies and the heterogeneity in trauma type, implant protocols, augmentation techniques, follow-up duration, and soft tissue outcome definitions. Because of this heterogeneity, quantitative meta-analysis was not feasible. Furthermore, long-term data (beyond three to five years) in trauma‐implant soft-tissue outcomes remain scarce. Future research should focus on prospective cohorts or randomized designs stratifying by trauma type (dentoalveolar vs. zygomatic/complex facial), defect characteristics, timing of implant placement, augmentation protocols, and use standardized esthetic‐soft tissue metrics (e.g., PES/WES) alongside patient‐reported outcomes.

## Conclusions

This systematic review highlights that oral and maxillofacial trauma does not inherently preclude favorable esthetic and soft tissue outcomes following dental implant rehabilitation. Across the included studies, immediate and delayed implant placement protocols both demonstrated stable peri-implant soft tissue levels and satisfactory esthetic scores when appropriate case selection, atraumatic surgical techniques, and adequate soft tissue management were implemented. The presence of trauma-related bone or soft tissue defects may, however, necessitate adjunctive regenerative or augmentation procedures to restore the pre-existing anatomy and optimize esthetic contours. Despite the generally positive outcomes reported, the available evidence remains limited by small sample sizes, heterogeneous study designs, and inconsistent methods of esthetic assessment. Most studies were observational in nature, with variable reporting of trauma characteristics and healing protocols, which restricts direct comparison and quantitative synthesis. Moreover, few investigations have incorporated patient-reported esthetic satisfaction or long-term soft tissue stability data, which are crucial for comprehensive evaluation of treatment success. Future research should focus on well-designed prospective studies with standardized assessment tools, such as the PES and WES, and long-term follow-up of post-traumatic implant cases. Particular attention should be given to the timing of implant placement after trauma, the influence of defect morphology, and the role of soft tissue augmentation procedures. Establishing evidence-based protocols for implant therapy in traumatized sites will aid clinicians in predicting esthetic outcomes and enhancing patient satisfaction in this complex clinical scenario.

## Appendices

Appendix A 

**Table 5:** Detailed search strategies for each database/register MEDLINE: Medical Literature Analysis and Retrieval System Online

Database/Register	Search string	Records
PubMed/MEDLINE	( "Maxillofacial Injuries"[MeSH] OR "Wounds and Injuries"[MeSH] OR "oral trauma" OR "maxillofacial trauma" OR "dentoalveolar trauma" OR "traumatic tooth loss" ) AND ( "Dental Implants"[MeSH] OR "implant placement" OR "immediate implant" OR "delayed implant" ) AND ( "Esthetics, Dental"[MeSH] OR "esthetic outcome" OR "soft tissue outcome" OR "pink esthetic score" OR "PES" OR "papilla height" OR "mucosal recession" )	223
ScienceDirect	(“oral trauma” OR “maxillofacial trauma” OR “dentoalveolar trauma”) AND (“dental implant”) AND (“esthetic outcomes” OR “soft tissue outcomes” OR “pink esthetic score”)	134
Cochrane Library	(oral trauma OR maxillofacial trauma OR dentoalveolar trauma) AND (dental implant*) AND (esthetic OR “soft tissue” OR “pink esthetic score” OR papilla OR recession)	17
Google Scholar	“oral trauma” AND “dental implant” AND “soft tissue esthetics” AND “pink esthetic score”	189
